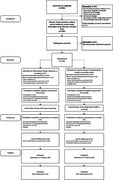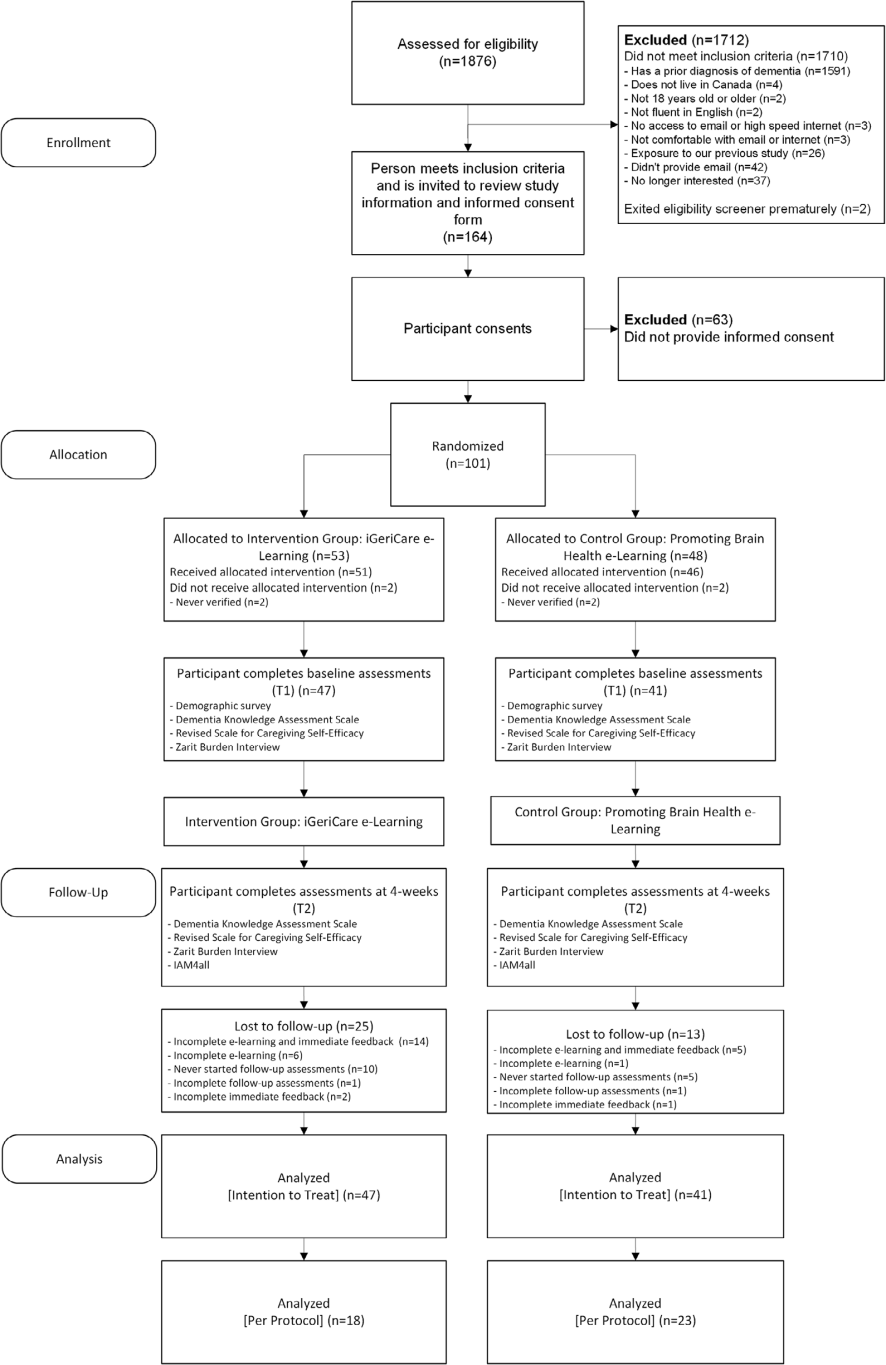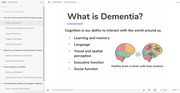# Feasibility and Acceptability of iGeriCare: A Pilot Randomized Controlled Trial of Web‐Based Dementia Education for Care Partners

**DOI:** 10.1002/alz70858_103426

**Published:** 2025-12-25

**Authors:** Anthony J. Levinson, Stephanie Ayers, Sandra Clark, Rebekah Woodburn, Maureen Markle‐Reid, Brian McKenna, Doug Oliver, Alexandra Papaioannou, Sarah Payne, Demetrios J. Sahlas, David Streiner, Dima Hadid, Amy Schneeberg, Henry Siu

**Affiliations:** ^1^ McMaster University, Hamilton, ON, Canada; ^2^ Independent Statistician, Vancouver, BC, Canada

## Abstract

**Background:**

Dementia is a significant global health challenge, with rising prevalence worldwide. Family and friend care partners play a critical role in supporting individuals with dementia but often lack adequate knowledge, leading to increased stress and burden. Web‐based interventions show promise in improving care partners’ mental health, yet few have been widely adopted or rigorously studied for knowledge outcomes. To address these gaps, iGeriCare was developed as a scalable dementia education platform, offering 12 multimedia lessons and email‐based micro‐learning to enhance care partner knowledge, self‐efficacy, and caregiving skills.

**Method:**

A two‐arm pilot feasibility randomized controlled trial (RCT) was conducted to evaluate feasibility, acceptance, and effectiveness among dementia care partners. Participants were randomized to either 1. iGeriCare e‐learning or 2. alternative e‐learning. Outcomes (adherence, study methods feasibility, participant feedback, dementia knowledge, self‐efficacy, and burden) were assessed at baseline and 8‐weeks. Challenges during the first recruitment cohort, including protocol deviations and fraudulent participation, informed refinements for a second cohort recruited via a paid panel. Qualitative thematic analysis of responses provided insights into barriers/facilitators, and care partner needs.

**Result:**

Cohort 1 (*n* = 125) began September 2022, with data collection concluding in January 2023. Cohort 2 (*n* = 100) was recruited in June 2023. Participants found the e‐learning content relevant, clear, and beneficial. They reported that the duration of the intervention was appropriate and the study methods acceptable. Preliminary results indicated significant knowledge gains in the intervention group among older care partners (≥45 years) with a mean increase of 13.9 points on a 50‐point scale compared to the control group. Self‐efficacy and burden differences between groups were not statistically significant. Thematic analysis revealed needs for skills in managing aggressive behaviors, communication, future care planning, emotional coping strategies for younger care partners (≤44), and practical caregiving strategies for older participants (≥45).

**Conclusion:**

This pilot study demonstrates the feasibility and acceptability of iGeriCare as a scalable, web‐based educational intervention for dementia care partners. Preliminary findings highlight the potential to improve knowledge and meet other needs. Lessons learned will inform the design of a larger RCT to evaluate long‐term impact on care partner outcomes, including knowledge self‐efficacy, and burden.